# Supplementary material for: Effects of the Quinone Oxidoreductase WrbA on Escherichia coli Biofilm Formation and Oxidative Stress
Source: Antioxidants (Basel). 2021 Jun 6;10(6):919. doi: 10.3390/antiox10060919 (PMC8229589; doi:10.3390/antiox10060919)
Supplement: Supplementary file 1 [file antioxidants-10-00919-s001.zip › Supplementary Figure 1.pdf]

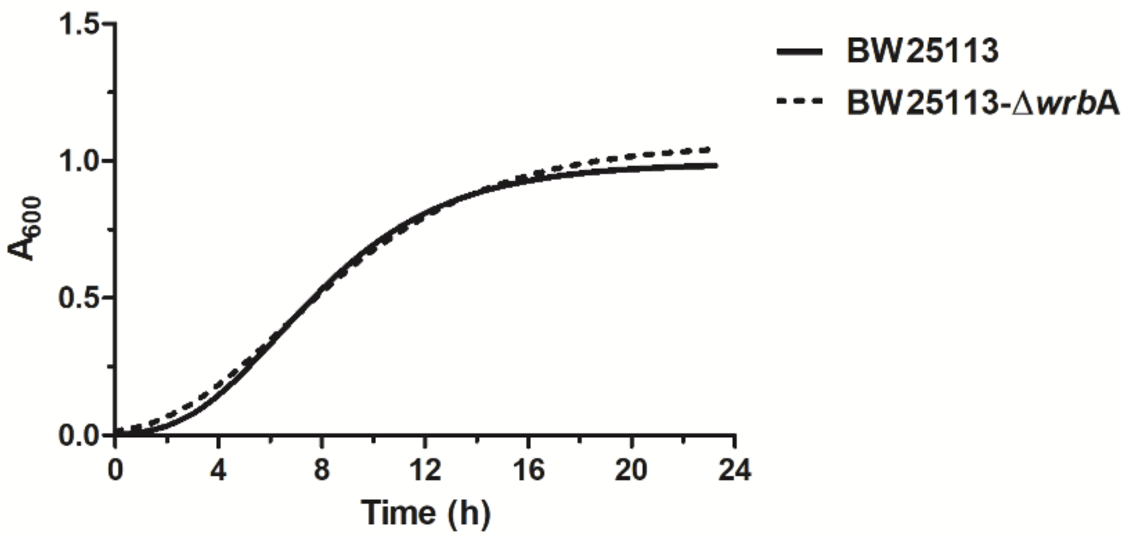

A

	BW25113	BW25113-ΔwrbA
λ (h)	2.75±0.27	2.13±0.48
µm (A_{600}/h)	0.10±0.003	0.09±0.01
Y_M (A_{600})	0.99±0.03	1.07±0.01
R^2	0.997±0.001	0.997±0.001

B

Supplementary Figure S1. BW25113 and BW25113-Δ*wrb*A planktonic growth. Panel A displays A_{600}-based growth curves, whereas panel B, the growth parameters of lag phase duration (λ), maximum specific growth rate (µm), maximum growth (Y_M) and the Goodness of Fit (R^2) obtained by the Gompertz model. Data represent the mean ± standard deviation of three independent measurements. No statistical differences were found among the strains.
