# Supplementary material for: Effects of the Quinone Oxidoreductase WrbA on Escherichia coli Biofilm Formation and Oxidative Stress
Source: Antioxidants (Basel). 2021 Jun 6;10(6):919. doi: 10.3390/antiox10060919 (PMC8229589; doi:10.3390/antiox10060919)
Supplement: Supplementary file 1 [file antioxidants-10-00919-s001.zip › Experimental data.pdf]

PLANKTONIC GROWTH CURVES

	replicate 1				replicate 2				replicate 3				replicate 1				replicate 2				replicate 3			
Time (h)	TSB wt	TSB wt	TSB wt	TSB wt	TSB wt	TSB wt	TSB wt	TSB wt	TSB wt	TSB wt	TSB wt	TSB wt	TSB wrbA-	TSB wrbA-	TSB wrbA-	TSB wrbA-	TSB wrbA-	TSB wrbA-	TSB wrbA-	TSB wrbA-	TSB wrbA-	TSB wrbA-	TSB wrbA-	TSB wrbA-
0	0,06196613	0,04266613	0,03506614	0,03116614	0,02736613	0,02756614	0,00696614	0,00506614	0,00636614	0,02186614	0,03096613	0,03226613	0,05316613	0,03666613	0,03626614	0,05026614	0,00496613	0,00066614	0,00166614	6,6133E-05	0,00176613	0,00426614	0,00396613	0,02586614
0,25	0,00156614	0,00056613	0,00226613	0,00276613	0,00216614	0,00196614	0,00276613	0,00146613	0,00176613	0,00256614	0,00126614	0,00206614	0,00196614	0,00126614	0,00196614	0,00146613	0,00066614	0,00016613	0,00056613	-0,0002339	0,00076614	0,00136613	0,00246614	0,00236613
0,5	0,00166614	0,00166614	0,00276613	0,00416614	0,00236613	0,00256614	0,00306613	0,00206614	0,00246614	0,00226613	0,00126614	0,00226613	0,00186613	0,00226613	0,00246614	0,00166614	0,00106614	0,00166614	0,00106614	0,00016613	0,00126614	0,00156614	0,00296614	0,00266613
0,75	0,00196614	0,00136613	0,00346614	0,00376614	0,00306613	0,00316613	0,00416614	0,00266613	0,00266613	0,00316613	0,00196614	0,00276613	0,00236613	0,00266613	0,00356613	0,00226613	0,00136613	0,00216614	0,00156614	0,00096613	0,00206614	0,00216614	0,00336614	0,00296614
1	0,00296614	0,00366613	0,00526613	0,00646614	0,00466614	0,00456613	0,00616613	0,00486613	0,00516614	0,00406613	0,00346614	0,00406613	0,00336614	0,00486613	0,00536613	0,00386614	0,00316613	0,00456613	0,00286614	0,00236613	0,00326614	0,00356613	0,00496613	0,00436614
1,25	0,00456613	0,00556614	0,00756613	0,00916614	0,00716613	0,00736614	0,00886613	0,00796613	0,00726614	0,00586613	0,00596614	0,00586613	0,00476614	0,00716613	0,00826614	0,00646614	0,00546614	0,00816614	0,00536613	0,00526613	0,00536613	0,00576613	0,00776614	0,00646614
1,5	0,00686614	0,00866614	0,01156613	0,01376613	0,01086614	0,01166613	0,01686613	0,01356614	0,01176614	0,00926613	0,01036614	0,00846613	0,00736614	0,01176614	0,01316614	0,01086614	0,01046614	0,01326613	0,01006614	0,01056613	0,00956614	0,00986614	0,01206614	0,00956614
1,75	0,01056613	0,01336613	0,01706614	0,02086613	0,01686613	0,01816613	0,02416614	0,02126613	0,01896613	0,01456613	0,01526614	0,01266614	0,01056613	0,01826614	0,02006614	0,01676613	0,01616614	0,02226614	0,01646613	0,01816613	0,01546613	0,01566614	0,01826614	0,01446614
2	0,01686613	0,02126613	0,02636614	0,03146613	0,02666614	0,02836614	0,03556613	0,03366613	0,03136613	0,02316614	0,02566613	0,02016614	0,01576614	0,02816613	0,03026614	0,02706614	0,02746613	0,03636613	0,02776613	0,02996613	0,02576613	0,02596613	0,02846614	0,02226614
2,25	0,02676614	0,03456613	0,04036614	0,04846614	0,04136614	0,04276613	0,05406613	0,05286614	0,05126613	0,03646613	0,04176613	0,03116614	0,02286613	0,04176613	0,04556613	0,04196613	0,04346613	0,05486613	0,04466613	0,04786613	0,04156614	0,04116613	0,04256613	0,03376614
2,5	0,04246613	0,05436613	0,06236614	0,07226614	0,06446613	0,06456613	0,07946613	0,08236613	0,08216613	0,05656613	0,06546613	0,04726613	0,03316613	0,06376613	0,06716613	0,06236614	0,06446613	0,08416613	0,06826613	0,07026614	0,06216613	0,06176614	0,06206613	0,05066613
2,75	0,06496613	0,08516613	0,09116613	0,1033661	0,09476613	0,09356613	0,1075661	0,1172661	0,1225661	0,08676613	0,09806613	0,06856614	0,04586614	0,09446613	0,09826613	0,09026613	0,09416614	0,1185661	0,09916613	0,1003661	0,09166614	0,09006613	0,08726614	0,07386614
3	0,09736613	0,1166661	0,1158661	0,1260661	0,1175661	0,1249661	0,1270661	0,1534661	0,1575661	0,1197661	0,1359661	0,09406614	0,06116613	0,1303661	0,1395661	0,1234661	0,1284661	0,1656661	0,1356661	0,1396661	0,1254661	0,1244661	0,1187661	0,1055661
3,25	0,1375661	0,1413661	0,1290661	0,1496661	0,1368661	0,1588661	0,1462661	0,1795661	0,1810662	0,1460661	0,1719661	0,1226661	0,08036613	0,1768661	0,1898662	0,1660661	0,1729662	0,2222661	0,1817662	0,1877661	0,1695662	0,1684662	0,1586661	0,1448661
3,5	0,1755661	0,1731662	0,1550661	0,1785661	0,1644661	0,1941662	0,1828661	0,2114661	0,2140661	0,1823661	0,2031661	0,1621661	0,1023661	0,2301661	0,2500661	0,2151662	0,2185661	0,2834661	0,2333661	0,2436661	0,2218661	0,2219661	0,2087661	0,1932662
3,75	0,2203662	0,2128661	0,1998661	0,2197661	0,2033661	0,2394661	0,2263661	0,2554661	0,2546661	0,2172661	0,2414661	0,2020662	0,1297661	0,2897661	0,3138661	0,2757661	0,2747661	0,3488661	0,2954662	0,3066661	0,2823661	0,2826661	0,2649661	0,2489661
4	0,2644661	0,2538661	0,2356662	0,2647661	0,2448661	0,2992661	0,2725661	0,3173662	0,3166661	0,2680661	0,2866662	0,2402661	0,1627661	0,3495661	0,3834662	0,3334661	0,3315662	0,4114662	0,3505661	0,3685662	0,3428662	0,3451661	0,3253661	0,3071661
4,25	0,3418661	0,3026661	0,2831661	0,3203661	0,2890661	0,3458661	0,3122661	0,3565661	0,3566661	0,3068661	0,3322662	0,2912661	0,1994661	0,4037662	0,4466662	0,3873661	0,3725661	0,4657662	0,3984661	0,4193662	0,3906661	0,3960661	0,3770661	0,3527661
4,5	0,3911662	0,3679661	0,3128662	0,3668661	0,3282661	0,3924661	0,3653661	0,4037662	0,3969661	0,3756661	0,3806661	0,3435661	0,2268662	0,4480661	0,4948662	0,4345662	0,4186662	0,5160661	0,4448662	0,4707662	0,4386661	0,4446661	0,4241661	0,3939661
4,75	0,4312662	0,4230662	0,3723661	0,4154662	0,4159661	0,4323662	0,4328661	0,4471661	0,4331661	0,4332661	0,4204662	0,4113661	0,2549661	0,4877661	0,5419661	0,4797662	0,4568661	0,5674661	0,4860661	0,5213661	0,4804662	0,4886661	0,4652661	0,4244662
5	0,4788661	0,4776662	0,4539662	0,4732662	0,4602661	0,4770661	0,4820661	0,4824661	0,4795661	0,4714662	0,4669661	0,4646662	0,2790661	0,5204661	0,5835661	0,5175661	0,4897662	0,6082661	0,5219661	0,5677661	0,5188661	0,5270661	0,5031661	0,4522661
5,25	0,5297661	0,5006661	0,4832661	0,4983661	0,4990661	0,5342661	0,5285661	0,5290661	0,5306661	0,5184661	0,5209661	0,5050661	0,2986661	0,5485661	0,6177661	0,5482661	0,5145661	0,6457661	0,5532661	0,6140661	0,5526661	0,5614661	0,5353661	0,4744661
5,5	0,5505661	0,5403661	0,5189661	0,5486661	0,5433661	0,5754661	0,5555661	0,5658661	0,5692661	0,5514661	0,5597661	0,5394661	0,3138661	0,5713661	0,6439661	0,5774661	0,5429661	0,6669661	0,5805661	0,6597661	0,5796661	0,5890661	0,5615661	0,4970662
5,75	0,5971661	0,5850661	0,5614661	0,5798661	0,5722661	0,5937661	0,5760661	0,5842661	0,5886661	0,5703661	0,5787661	0,5725661	0,3276661	0,5960661	0,6662661	0,6028661	0,5721661	0,6823661	0,6042661	0,6943661	0,6055661	0,6086661	0,5832661	0,5174661
6	0,6259661	0,6082661	0,5826661	0,5960661	0,5879661	0,6106661	0,5919661	0,6018661	0,6061661	0,5845661	0,5954661	0,5927661	0,3452661	0,6182661	0,6841661	0,6242661	0,5976661	0,6941661	0,6211661	0,7179661	0,6243661	0,6236661	0,5969661	0,5301661
6,25	0,6460661	0,6271661	0,6010661	0,6132661	0,6017661	0,6264661	0,6066661	0,6176661	0,6223661	0,6004661	0,6114661	0,6078661	0,3527661	0,6332661	0,6967661	0,6388661	0,6255661	0,7029661	0,6378661	0,7414661	0,6387661	0,6328661	0,6044661	0,5385661
6,5	0,6680661	0,6413661	0,6196661	0,6255661	0,6169661	0,6381661	0,6197661	0,6295661	0,6359661	0,6133661	0,6237661	0,6222661	0,3558661	0,6454661	0,7092661	0,6512661	0,6395661	0,7087661	0,6528661	0,7602661	0,6534661	0,6428661	0,6119661	0,5429661
6,75	0,6807661	0,6553661	0,6336661	0,6362661	0,6283661	0,6490661	0,6337661	0,6414661	0,6478661	0,6276661	0,5281661	0,6369661	0,3615661	0,6564661	0,7203661	0,6629661	0,6539661	0,7217661	0,6632661	0,7745661	0,6632661	0,6511661	0,6208661	0,5464661
7	0,6908661	0,6645661	0,6461661	0,6462661	0,6367661	0,6573661	0,6441661	0,6506661	0,6572661	0,6379661	0,5354661	0,6473661	0,3632661	0,6629661	0,7294661	0,6700661	0,6655661	0,7293661	0,6718661	0,7890661	0,6725661	0,6597661	0,6262661	0,5496661
7,25	0,6989661	0,6747661	0,6587661	0,6570661	0,6458661	0,6661661	0,6546661	0,6608661	0,6667661	0,6483661	0,5907661	0,6601661	0,3666661	0,6710661	0,7388661	0,6780661	0,6775661	0,7443661	0,6793661	0,8032661	0,6808661	0,6678661	0,6323661	0,5530661
7,5	0,7049661	0,6824661	0,6705661	0,6662661	0,6520661	0,6736661	0,6635661	0,6676661	0,6752661	0,6572661	0,6273661	0,6683661	0,3739661	0,6742661	0,7452661	0,6840661	0,6887661	0,7525661	0,6863661	0,8124661	0,6882661	0,6742661	0,6384661	0,5559661
7,75	0,7158661	0,6920661	0,6806661	0,6749661	0,6618661	0,6820661	0,6720661	0,6759661	0,6845661	0,6671661	0,6563661	0,6788661	0,3814662	0,6809661	0,7522661	0,6932661	0,7005661	0,7629661	0,6959661	0,8310661	0,6997661	0,6821661	0,6462661	0,5644661
8	0,7270661	0,7054661	0,6965661	0,6877661	0,6721661	0,6944661	0,6844661	0,6887661	0,6972661	0,6794661	0,6814661	0,6912661	0,3858661	0,6842661	0,7645661	0,6979661	0,7125661	0,7714661	0,7031661	0,8400661	0,7065661	0,6886661	0,6527661	0,5644661
8,25	0,7343661	0,7122661	0,7031661	0,6921661	0,6767661	0,6974661	0,6877661	0,6934661	0,7016661	0,6850661	0,6938661	0,6977661	0,3903661	0,6882661	0,7691661	0,7044661	0,7195661	0,7770661	0,7089661	0,8489661	0,7132661	0,6956661	0,6593661	0,5685661
8,5	0,7445661	0,7203661	0,7113661	0,6976661	0,6838661	0,7050661	0,6946661	0,7001661	0,7083661	0,6922661	0,7030661	0,7058661	0,3996661	0,6940661	0,7788661	0,7155661	0,7341661	0,7829661	0,7213661	0,8697661	0,7269661	0,7042661	0,6676661	0,5758661
8,75	0,7535661	0,7236661	0,7151661	0,6982661	0,6867661	0,7075661	0,6968661	0,7036661	0,7096661	0,6953661	0,7075661	0,7127661	0,4070661	0,6973661	0,7896661	0,7231661	0,7414661	0,7934661	0,7290661	0,8758661	0,7316661	0,7104661	0,6753661	0,5761661
9	0,7571661	0,7220661	0,7136661	0,6965661	0,6868661	0,7067661	0,6976661	0,7034661	0,7097661	0,6960661	0,7083661	0,7150661	0,4131661	0,7032661	0,7957661	0,7297661	0,7527661	0,8048661	0,7371661	0,8850661	0,7411661	0,7201661	0,6830661	0,5811661
9,25	0,7670661	0,7268661	0,7183661	0,7023661	0,6937661	0,7138661	0,7030661	0,7080661	0,7139661	0,7005661	0,7116661	0,7204661	0,4207661	0,7080661	0,8056661	0,7382661	0,7570661	0,8169661	0,7444661	0,8984661	0,7496661	0,7270661	0,6898661	0,5886661
9,5	0,7670661	0,7247661	0,7168661	0,7016661	0,6966661	0,7160661	0,7066661	0,7101661	0,7162661	0,7029661	0,7131661	0,7234661	0,4322661	0,7139661	0,8147661	0,7462661	0,7564661	0,8240661	0,7516661	0,9079661	0,7577661	0,7353661	0,6977661	0,5930661
9,75	0,7667661	0,7253661	0,7181661	0,7036661	0,7013661	0,7198661	0,7108661	0,7137661	0,7200661	0,7055661	0,7166661	0,7285661	0,4419661	0,7199661	0,8202661	0,7525661	0,7561661	0,8413661	0,7615661	0,9181662	0,7674661	0,7461661	0,7091661	0,5995661
10	0,7790661	0,7318661	0,7254661	0,7104661	0,7103661	0,7275661	0,7173661	0,7200661	0,7264661	0,7112661	0,7230661	0,7374661	0,4507661	0,7244661	0,8289661	0,7582661	0,7530661	0,8566661	0,7684661	0,9279661	0,7739661	0,7535661	0,7168661	0,6031661
10,25	0,7889661	0,7404661	0,7332661	0,7183661	0,7204661	0,7362661	0,7247661	0,7265661	0,7337661	0,7175661	0,7307661	0,7467661	0,4612661	0,7309661	0,8397661	0,7672661	0,7548661	0,8769661	0,7780661	0,9398661	0,7855661	0,7639661	0,7258661	0,6108661
10,5	0,7919661	0,7451661	0,7383661	0,7242661	0,7291661	0,7451661	0,7319661	0,7328661	0,7405661	0,7242661	0,7400661	0,7530661	0,4722662	0,7383661	0,8485661	0,7778661	0,7596661	0,9042661	0,7883661	0,9523661	0,8022661	0,7798661	0,7386661	0,6215661
10,75	0,7982661	0,7525661	0,7444661	0,7310661	0,7379661	0,7524661	0,7387661	0,7395661	0,7468661	0,7299661	0,7496661	0,7592661	0,4853661	0,7441661	0,8541661	0,7847661	0,7655661	0,9030661	0,7960661	0,9577661	0,8137661	0,7911661	0,7490661	0,6266661
11	0,8141661	0,7638661	0,7534661	0,7399661	0,7493661	0,7636661	0,7476661	0,7476661	0,7549661	0,7372661	0,7588661	0,7696661	0,4962662	0,7489661	0,8676661	0,7939661	0,7732661	0,9210662	0,8047661	0,9671661	0,8198661	0,8022661	0,7587661	0,6345661
11,25	0,8178661	0,7742661	0,7618661	0,7495661	0,7592661	0,7743661	0,7573661	0,7574661	0,7634661	0,7480661	0,7698661	0,7774661	0,5080661	0,7526661	0,8820661	0,8013661	0,7793661	0,9633662	0,8144661	0,9711661	0,8330661	0,8151661	0,7706661	0,6428661
11,5	0,8222661	0,7791661	0,7678661	0,7558661	0,7692661	0,7812661	0,7644661	0,7627661	0,7689661	0,7548661	0,7781661	0,7779661	0,5212661	0,7569661	0,8912661	0,8093661	0,7877661	0,9642661	0,8254661	0,9779661	0,8463661	0,8278661	0,7856661	0,6507661
11,75	0,8323661	0,7856661	0,7734661	0,7616661	0,7779661	0,7893661	0,7713661	0,7696661	0,7760661	0,7615661	0,7877661	0,7909661	0,5326661	0,7631661	0,9016661	0,8196661	0,7971661	0,9668661	0,8371661	0,9876661	0,8605661	0,8393661	0,7978661	0,6577661
12	0,8368661	0,7905661	0,7790661	0,7678661	0,7860661	0,7960661	0,7779661	0,7749661	0,7817661	0,7672661	0,7954661	0,7975661	0,5420661	0,7653661	0,9110661	0,8303661	0,8095661	0,9721661	0,8477661	0,9935662	0,8726661	0,8532661	0,8110661	0,6656661
12,25	0,8499661	0,7989661	0,7870661	0,7761661	0,7945661	0,8040661	0,7865661	0,7837661	0,7910661	0,7753661	0,8041661	0,8068661	0,5528661	0,7696661	0,9230661	0,8426661	0,8215661	1,008866	0,8616661	1,003866	0,8868661	0,8674661	0,8250661	0,6737661
12,5	0,8555661	0,8063661	0,7942661	0,7830661	0,8017661	0,8105661	0,7933661	0,7903661	0,7979661	0,7826661	0,8111661	0,8118661	0,5587661	0,7750661	0,9331661	0,8551661	0,8298661	1,011966	0,8755661	1,012766	0,9006661	0,8821661	0,8409661	0,6834661
12,75	0,8679661	0,8166661	0,8038661	0,7929661	0,8116661	0,8199661	0,8032661	0,8003661	0,8081661	0,7920661	0,8197661	0,8194661	0,5682661	0,7790661	0,9515661	0,8664661	0,8391661	1,015166	0,8874661	1,022366	0,9141661	0,8955661	0,8562661	0,6908661
13	0,8754661	0,8256661	0,8126661	0,8008661	0,8185661	0,8276661	0,8117661	0,8092661	0,8161661	0,8014661	0,8285661	0,8228661	0,5757661	0,7850661	0,9635661	0,8785661	0,8529661	1,038266	0,8993661	1,029866	0,9291661	0,9111661	0,8706661	0,6986661
13,25	0,8763661	0,8315661	0,8199661	0,8080661	0,8251661	0,8336661	0,8191661	0,8175661	0,8243661	0,8095661	0,8352661	0,8259661	0,5830661	0,7939661	0,9750661	0,8915661	0,8647661	1,055866	0,9142661	1,038966	0,9431661	0,9260662	0,8867661	0,7099661
13,5	0,8846661	0,8397661	0,8270661	0,8145661	0,8314661	0,8413661	0,8277661	0,8253661	0,8318661	0,8177661	0,8407661	0,8300661	0,5912661	0,8043661	0,9910661	0,9065661	0,8783661	1,053966	0,9287661	1,046966	0,9582661	0,9405661	0,9007661	0,7175661
13,75	0,8893661	0,8456661	0,8337661	0,8215661	0,8365661	0,8471661	0,8346661	0,8323661	0,8387661	0,8245661	0,8467661	0,8344661	0,5998661	0,8130661	1,003166	0,9200661	0,8924661	1,037366	0,9448661	1,053866	0,9736661	0,9569662	0,9184661	0,7278661
14	0,8974661	0,8521661	0,8414661	0,8288661	0,8442661	0,8536661	0,8412661	0,8398661	0,8466661	0,8327661	0,8524661	0,8413661	0,6133661	0,8254661	1,019066	0,9370661	0,9049661	1,029966	0,9610661	1,062666	0,9876661	0,9718661	0,9348661	0,7422661
14,25	0,9022661	0,8582661	0,8478661	0,8355661	0,8510661	0,8608661	0,8476661	0,8462661	0,8528661	0,8403661	0,8584661	0,8472661	0,6213661	0,8364661	1,035066	0,9516661	0,9192661	1,022566	0,9754661	1,067866	1,000066	0,9858661	0,9491661	0,7549661
14,5	0,9068661	0,8634661	0,8544661	0,8410661	0,8558661	0,8655661	0,8531661	0,8523661	0,8574661	0,8469661	0,8643661	0,8539661	0,6314661	0,8498661	1,046166	0,9647661	0,9315661	1,016966	0,9890661	1,074966	1,013366	1,000966	0,9661661	0,7629661
14,75	0,9145662	0,8703661	0,8613661	0,8480661	0,8634661	0,8726661	0,8618661	0,8606661	0,8654661	0,8549661	0,8706661	0,8619661	0,6427661	0,8627661	1,058166	0,9759661	0,9423661	1,004866	1,000166	1,081266	1,023966	1,013666	0,9810661	0,7744661
15	0,9179661	0,8757661	0,8671661	0,8536661	0,8687661	0,8784661	0,8683661	0,8675661	0,8710661	0,8600661	0,8762661	0,8676661	0,6549661	0,8783661	1,068966	0,9901661	0,9550661	1,013866	1,011766	1,088266	1,036266	1,026166	0,9946661	0,7941661
15,25	0,9260662	0,8835661	0,8745661	0,8613661	0,8745661	0,8843661	0,8731661	0,8724661	0,8767661	0,8651661	0,8810661	0,8747661	0,6667661	0,8927661	1,077466	1,000866	0,9635661	1,023866	1,022466	1,094166	1,043966	1,036066	1,005966	0,8075661
15,5	0,9296662	0,8881661	0,8789661	0,8664661	0,8803661	0,8897661	0,8793661	0,8782661	0,8827661	0,8725661	0,8863661	0,8820661	0,6835661	0,9085661	1,089266	1,010766	0,9754661	1,028366	1,033766	1,100166	1,053966	1,046866	1,019166	0,8276661
15,75	0,9386661	0,8961661	0,8865661	0,8737661	0,8874661	0,8973661	0,8865661	0,8852661	0,8891661	0,8774661	0,8912661	0,8903661	0,6991661	0,9217662	1,098066	1,020666	0,9842662	1,030966	1,041766	1,104266	1,061766	1,055566	1,028266	0,8380661
16	0,9451661	0,9031661	0,8933661	0,8794661	0,8932661	0,9034661	0,8932661	0,8917661	0,8953661	0,8825661	0,8968661	0,8964661	0,7189661	0,9362661	1,105366	1,028766	0,9963661	1,033666	1,050866	1,109366	1,069866	1,064566	1,038266	0,8561661
16,25	0,9505661	0,9091661	0,8999661	0,8869661	0,8996661	0,9103661	0,9002661	0,8979661	0,9022661	0,8902661	0,9045661	0,9033661	0,7379661	0,9489661	1,113466	1,037666	1,005966	1,034466	1,058066	1,115566	1,076966	1,072366	1,046966	0,8779661
16,5	0,9585661	0,9158661	0,9060661	0,8904661	0,9043661	0,9154661	0,9059661	0,9039661	0,9077661	0,8962661	0,9103661	0,9104661	0,7589661	0,9594661	1,122166	1,047366	1,015566	1,028566	1,067566	1,119866	1,084366	1,080966	1,057166	0,8947661
16,75	0,9599661	0,9202662	0,9114661	0,8989661	0,9102662	0,9215661	0,9126661	0,9107661	0,9148661	0,9018661	0,9162661	0,9142661	0,7815661	0,9704661	1,128966	1,055166	1,026166	1,026166	1,074866	1,123866	1,091066	1,088666	1,065566	0,9135661
17	0,9643661	0,9252661	0,9167662	0,9039661	0,9154661	0,9262661	0,9175661	0,9171661	0,9205661	0,9090661	0,9220661	0,9206661	0,8081661	0,9809661	1,135966	1,063366	1,035966	1,033766	1,082066	1,128266	1,097766	1,095466	1,073666	0,9351661
17,25	0,9719661	0,9314661	0,9226661	0,9081661	0,9213661	0,9320661	0,9249661	0,9227661	0,9270661	0,9143661	0,9278661	0,9263661	0,8277661	0,9892662	1,140866	1,072266	1,043366	1,045666	1,089166	1,133766	1,105266	1,103366	1,081266	0,9509661
17,5	0,9751661	0,9364661	0,9293661	0,9164661	0,9268661	0,9373661	0,9299661	0,9287661	0,9336661	0,9207661	0,9324661	0,9318662	0,8523661	0,9983661	1,148466	1,079666	1,053466	1,050966	1,095966	1,136966	1,109966	1,109666	1,089266	0,9677662
17,75	0,9820662	0,9423661	0,9346662	0,9221661	0,9334661	0,9429661	0,9360662	0,9354662	0,9399661	0,9269661	0,9381661	0,9370661	0,8679661	1,008966	1,153566	1,086766	1,060566	1,056666	1,103066	1,141166	1,115966	1,116166	1,096666	0,9829661
18	0,9882661	0,9485661	0,9399661	0,9277661	0,9383661	0,9480661	0,9428661	0,9412661	0,9454662	0,9332662	0,9439662	0,9427661	0,8878661	1,017766	1,159166	1,093766	1,069666	1,062866	1,107566	1,144166	1,121366	1,121866	1,102666	0,9950662
18,25	0,9951661	0,9548661	0,9465661	0,9349661	0,9439662	0,9546661	0,9487661	0,9480661	0,9530661	0,9398661	0,9500661	0,9498661	0,9113661	1,027566	1,165266	1,101466	1,079166	1,061766	1,113366	1,146966	1,126266	1,127666	1,109866	1,011166
18,5	0,9999661	0,9606661	0,9531661	0,9412661	0,9501661	0,9607661	0,9545661	0,9536661	0,9591662	0,9458661	0,9552661	0,9548661	0,9281662	1,035266	1,168866	1,106666	1,084466	1,065366	1,118566	1,150866	1,130866	1,131966	1,114966	1,022266
18,75	1,008266	0,9674661	0,9595661	0,9475662	0,9571661	0,9682661	0,9622661	0,9603661	0,9655662	0,9530661	0,9621661	0,9609661	0,9414661	1,039766	1,173766	1,112966	1,090666	1,085266	1,124466	1,153866	1,135966	1,137966	1,120566	1,033066
19	1,010166	0,9723661	0,9660661	0,9549661	0,9620661	0,9722661	0,9676661	0,9663661	0,9713662	0,9585661	0,9673661	0,9641662	0,9594661	1,051366	1,179266	1,119166	1,100766	1,137466	1,128766	1,156766	1,139366	1,142666	1,127066	1,045166
19,25	1,014666	0,9779661	0,9700661	0,9589661	0,9674661	0,9782661	0,9744661	0,9727662	0,9759661	0,9635661	0,9731661	0,9690661	0,9742661	1,059466	1,183666	1,125966	1,108666	1,156766	1,133366	1,159066	1,143866	1,146966	1,130966	1,054466
19,5	1,020266	0,9829661	0,9773661	0,9652661	0,9735661	0,9841661	0,9794661	0,9778661	0,9811661	0,9686661	0,9776661	0,9735661	0,9867661	1,066166	1,186366	1,130966	1,113366	1,160666	1,138366	1,160966	1,147366	1,151166	1,136266	1,063166
19,75	1,024266	0,9883661	0,9824661	0,9715661	0,9791662	0,9898661	0,9864662	0,9840661	0,9869661	0,9767661	0,9840661	0,9796661	0,9985662	1,076066	1,190466	1,136166	1,118566	1,167566	1,143066	1,166166	1,152266	1,156066	1,141766	1,072266
20	1,031266	0,9954661	0,9874661	0,9773661	0,9848661	0,9954661	0,9925661	0,9901661	0,9925661	0,9821661	0,9899662	0,9855661	1,008266	1,079866	1,193366	1,140366	1,123266	1,155466	1,145966	1,165666	1,154366	1,159566	1,145666	1,079866
20,25	1,036266	1,002166	0,9948661	0,9840661	0,9918661	1,000766	0,9991661	0,9957662	0,9991661	0,9884661	0,9951661	0,9904661	1,016566	1,087666	1,195766	1,145266	1,128166	1,150066	1,150966	1,168766	1,158666	1,164266	1,151066	1,087966
20,5	1,041266	1,007066	0,9988661	0,9886661	0,9965661	1,005866	1,003766	1,000866	1,003866	0,9936661	0,9995661	0,9952661	1,025466	1,096466	1,199866	1,150666	1,133466	1,154566	1,154366	1,170966	1,161566	1,167766	1,155466	1,095166
20,75	1,047266	1,011666	1,003566	0,9919661	1,000866	1,011466	1,008466	1,006166	1,008166	0,9985662	1,005066	1,001366	1,033966	1,101466	1,202666	1,154166	1,138866	1,149166	1,157566	1,172666	1,164166	1,171066	1,158266	1,100766
21	1,051866	1,016666	1,008966	0,9980661	1,006366	1,016566	1,013566	1,011166	1,014366	1,003766	1,010266	1,006866	1,041866	1,108266	1,204966	1,158066	1,142366	1,150366	1,161366	1,174966	1,167166	1,174766	1,162966	1,106766
21,25	1,057766	1,021966	1,014266	1,002366	1,010966	1,022166	1,019066	1,016266	1,019266	1,008966	1,015166	1,011866	1,048666	1,113066	1,207366	1,163266	1,145166	1,159366	1,163966	1,176466	1,170466	1,177866	1,166466	1,113266
21,5	1,062466	1,025866	1,018566	1,006966	1,015966	1,026066	1,023266	1,021166	1,023566	1,014266	1,020766	1,017166	1,057066	1,118566	1,210666	1,165366	1,147966	1,167666	1,167066	1,178466	1,172966	1,180866	1,170166	1,118166
21,75	1,067666	1,030866	1,024066	1,013566	1,021066	1,031966	1,028566	1,026066	1,028666	1,019666	1,025866	1,022866	1,063166	1,126066	1,212466	1,169966	1,151766	1,183566	1,170666	1,181566	1,175666	1,183966	1,173866	1,123866
22	1,074166	1,036866	1,029366	1,018666	1,027466	1,037366	1,033966	1,031666	1,033766	1,024966	1,032166	1,029466	1,066666	1,129466	1,213666	1,172666	1,154766	1,178866	1,172566	1,181966	1,178866	1,186666	1,176966	1,128166
22,25	1,080366	1,042466	1,035266	1,024266	1,033566	1,040366	1,039266	1,035966	1,037966	1,029666	1,035966	1,035766	1,070366	1,131866	1,213966	1,173566	1,155366	1,159166	1,175466	1,183766	1,180566	1,190066	1,180166	1,133266
22,5	1,086466	1,047666	1,039966	1,030166	1,038866	1,046166	1,044366	1,040566	1,042566	1,035266	1,042166	1,040666	1,076466	1,137366	1,217066	1,178666	1,157966	1,158566	1,178166	1,185366	1,183466	1,192166	1,182566	1,137666
22,75	1,091266	1,053066	1,045666	1,034266	1,044666	1,050066	1,049066	1,045066	1,047866	1,039366	1,046766	1,046066	1,079166	1,140966	1,218166	1,180866	1,160566	1,153766	1,180366	1,186466	1,185166	1,194166	1,185866	1,142266
23	1,097866	1,058466	1,051266	1,039766	1,049166	1,055666	1,053866	1,049166	1,051366	1,044266	1,050566	1,051166	1,084266	1,144066	1,220066	1,182666	1,161766	1,152266	1,182366	1,188066	1,187366	1,196766	1,188566	1,145366
23,25	1,101166	1,062166	1,055166	1,043666	1,053166	1,058466	1,057666	1,053566	1,054066	1,047966	1,055066	1,055166	1,089266	1,147566	1,220666	1,184866	1,163466	1,166366	1,184766	1,189566	1,189366	1,199366	1,190866	1,149766

Gompertz Be	YM	B	C	R²	l (h)	l average (h)	l sd	l err %		µm (OD600/	µm average	µm sd	µm err %		YM (OD600)	µm average	µm sd	µm err %		R2	R2 average	sd	err %
LB wt	1,007	1,914	0,282	0,9976	3,24113475	2,74683314	0,26836177	9,76986054		0,1044682	0,1024028	0,0026291	2,56740668		1,007	0,99197273	0,02514299	2,53464576		0,9976	0,99717273	0,00109461	0,10977185
LB wt	0,9668	1,708	0,2773	0,9967	2,55319149					0,09862614					0,9668					0,9967			
LB wt	0,9687	1,89	0,2921	0,9955	3,04690175					0,10409416					0,9687					0,9955			
LB wt	0,9739	1,722	0,2806	0,9959	2,57305773					0,10053275					0,9739					0,9959			
LB wt	1,039	1,672	0,2806	0,9963	2,39486814					0,10725282					1,039					0,9963			
LB wt	0,9852	1,851	0,2893	0,9967	2,94158313					0,1048524					0,9852					0,9967			
LB wt	0,9998	1,677	0,2735	0,9981	2,47531993					0,1005949					0,9998					0,9981			
LB wt	0,9715	1,827	0,2853	0,9973	2,89870312					0,10196476					0,9715					0,9973			
LB wt	0,9798	1,698	0,2748	0,9972	2,54002911					0,09905119					0,9798					0,9972			
LB wt	0,987	1,808	0,2823	0,9988	2,86220333					0,10250229					0,987					0,9988			
LB wt	1,033	1,725	0,2697	0,9988	2,68817204					0,10249125					1,033					0,9988			
LB wrbA-	1,091	1,455	0,1968	0,9946	2,31199187	2,12945118	0,48308681	22,6859772		0,07898695	0,08909493	0,00514673	5,77667755		1,091	1,06716667	0,01294628	1,21314471		0,9946	0,997175	0,00154692	0,15513002
LB wrbA-	1,051	1,482	0,2439	0,9979	1,97621976					0,09430181					1,051					0,9979			
LB wrbA-	1,067	1,315	0,212	0,994	1,48584906					0,0832158					1,067					0,994			
LB wrbA-	1,062	1,385	0,2317	0,9961	1,66163142					0,0905224					1,062					0,9961			
LB wrbA-	1,059	1,459	0,2269	0,9978	2,02291758					0,08839668					1,059					0,9978			
LB wrbA-	1,073	1,446	0,2288	0,9976	1,9493007					0,09031529					1,073					0,9976			
LB wrbA-	1,087	1,417	0,2243	0,9969	1,85911725					0,08969419					1,087					0,9969			
LB wrbA-	1,074	1,529	0,2359	0,9985	2,24247563					0,09320468					1,074					0,9985			
LB wrbA-	1,059	1,723	0,2104	0,9972	3,43631179					0,08196854					1,059					0,9972			
LB wrbA-	1,057	1,509	0,2412	0,9984	2,11028192					0,09379027					1,057					0,9984			
LB wrbA-	1,052	1,569	0,245	0,9989	2,32244898					0,09481725					1,052					0,9989			
LB wrbA-	1,074	1,495	0,2276	0,9982	2,17486819					0,08992533					1,074					0,9982			
TSB wt	1,001	1,5	0,3208	0,9717	1,55860349	1,23014865	0,19708462	16,0212039		0,11813374	0,10335881	0,00602437	5,82860176		1,001	0,96688333	0,01270518	1,3140341		0,9717	0,96785833	0,00264625	0,27341319
TSB wt	0,9644	1,425	0,3057	0,9671	1,39025188					0,10845714					0,9644					0,9671			
TSB wt	0,9617	1,418	0,2954	0,9697	1,41503047					0,10450947					0,9617					0,9697			
TSB wt	0,9497	1,332	0,2877	0,965	1,15397984					0,10051522					0,9497					0,965			
TSB wt	0,9665	1,352	0,2807	0,9694	1,25400784					0,09980442					0,9665					0,9694			
TSB wt	0,9687	1,341	0,2921	0,967	1,16740842					0,10409416					0,9687					0,967			
TSB wt	0,9697	1,297	0,2761	0,9664	1,07569721					0,0984939					0,9697					0,9664			
TSB wt	0,9642	1,265	0,2785	0,9648	0,95152603					0,09878656					0,9642					0,9648			
TSB wt	0,9655	1,29	0,2851	0,9662	1,01718695					0,10126398					0,9655					0,9662			
TSB wt	0,9575	1,327	0,2817	0,9648	1,16080937					0,09922729					0,9575					0,9648			
TSB wt	0,9762	1,3	0,2722	0,9722	1,10213079					0,09775353					0,9762					0,9722			
TSB wt	0,9575	1,47	0,3102	0,97	1,51515152					0,10926626					0,9575					0,97			
TSB wrbA-	1,188	1,206	0,2455	0,9689	0,83910387	0,89571083	0,32114648	35,8538122		0,10729351	0,1038415	0,02023124	19,4828078		1,188	1,16	0,03198828	2,75761027		0,9689	0,97175556	0,00594225	0,61149588
TSB wrbA-	1,158	1,147	0,2154	0,9667	0,68245125					0,09176135					1,158					0,9667			
TSB wrbA-	1,128	1,157	0,2198	0,9653	0,71428571					0,09120997					1,128					0,9653			
TSB wrbA-	1,105	1,365	0,3114	0,9741	1,17212588					0,12658621					1,105					0,9741			
TSB wrbA-	1,163	1,165	0,2214	0,9707	0,74525745					0,09472462					1,163					0,9707			
TSB wrbA-	1,136	1,573	0,3512	0,9854	1,63154897					0,14677036					1,136					0,9854			
TSB wrbA-	1,17	1,211	0,2293	0,9746	0,92019189					0,09869506					1,17					0,9746			
TSB wrbA-	1,19	1,157	0,212	0,9705	0,74056604					0,09280863					1,19					0,9705			
TSB wrbA-	1,202	1,118	0,1916	0,9696	0,61586639					0,08472381					1,202					0,9696			

BUIOFILM GROWTH CURVES

BW25113

	time	CFU/cm2	N/N0	log10(N/N0)		time	avarage	stand dev
replicate 1	0	2,17E+08	1,00E+00	0		0	0,00	0,00
replicate 2	0	2,17E+08	1,00E+00	0,00		5	0,86	0,06
replicate 3	0	1,68E+08	7,75E-01	-0,11		12	2,10	0,13
replicate 1	5	1,63E+09	7,51E+00	0,88		18	2,34	0,70
replicate 2	5	1,83E+09	8,45E+00	0,93		24	2,47	0,04
replicate 3	5	1,29E+09	5,95E+00	0,77		36	3,07	0,11
replicate 1	12	1,79E+10	8,26E+01	1,92		48	3,96	0,03
replicate 2	12	3,37E+10	1,55E+02	2,19		60	8,86	0,16
replicate 3	12	3,42E+10	1,58E+02	2,20		72	9,76	0,03
replicate 1	18	1,46E+10	6,72E+01	1,83				
replicate 2	18	1,60E+10	7,36E+01	1,87				
replicate 3	18	4,64E+11	2,14E+03	3,33				
replicate 1	24	6,52E+10	3,00E+02	2,48				
replicate 2	24	5,64E+10	2,60E+02	2,41				
replicate 3	24	6,97E+10	3,21E+02	2,51				
replicate 1	36	1,83E+11	8,42E+02	2,93				
replicate 2	36	3,19E+11	1,47E+03	3,17				
replicate 3	36	2,93E+11	1,35E+03	3,13				
replicate 1	48	1,90E+12	8,76E+03	3,94				
replicate 2	48	2,21E+12	1,02E+04	4,01				
replicate 3	48	1,90E+12	8,76E+03	3,94				
replicate 1	60	2,28E+17	1,05E+09	9,02				
replicate 2	60	1,08E+17	5,00E+08	8,70				
replicate 1	72	1,15E+18	5,31E+09	9,72				
replicate 2	72	1,36E+18	6,28E+09	9,80				
replicate 3	72	1,22E+18	5,63E+09	9,75				

BW25113-Δ*wrb* A

	time	CFU/cm2	N/N0	log10(N/N0)		time	avarage	stand dev
replicate 1	0	9,37E+07	1,00E+00	0,00		0	-0,03	0,03
replicate 2	0	8,06E+07	8,60E-01	-0,07		5	1,20	0,09
replicate 3	0	1,61E+08	1,72E+00	0,23		12	3,79	0,08
replicate 1	5	1,22E+09	1,30E+01	1,11		18	6,24	0,04
replicate 2	5	1,98E+09	2,11E+01	1,32		24	5,07	0,08
replicate 3	5	1,40E+09	1,49E+01	1,17		36	5,85	0,00
replicate 1	12	4,77E+11	5,09E+03	3,71		48	6,33	0,00
replicate 2	12	6,91E+11	7,38E+03	3,87		60	7,51	0,13
replicate 3	12					72	7,71	0,04
replicate 1	18	1,71E+14	1,83E+06	6,26				
replicate 2	18	1,43E+14	1,52E+06	6,18				
replicate 3	18	1,75E+14	1,87E+06	6,27				
replicate 1	24	1,07E+13	1,14E+05	5,06				
replicate 2	24	1,39E+13	1,48E+05	5,17				
replicate 3	24	8,83E+12	9,42E+04	4,97				
replicate 1	36	6,58E+13	7,03E+05	5,85				
replicate 2	36	6,72E+13	7,17E+05	5,86				
replicate 3	36							
replicate 1	48	2,02E+14	2,15E+06	6,33				
replicate 2	48	2,00E+14	2,13E+06	6,33				
replicate 3	48							
replicate 1	60	4,12E+15	4,40E+07	7,64				
replicate 2	60	2,24E+15	2,39E+07	7,38				
replicate 1	72	5,13E+15	5,48E+07	7,74				
replicate 2	72	5,22E+15	5,57E+07	7,75				
replicate 3	72	4,16E+15	4,43E+07	7,65				

BIOFILM THICKNESS				
BW25113 (um)			BW25113-ΔwrbA (um)	
replicate 1	228		replicate 1	149
replicate 2	224		replicate 2	115
replicate 3	247		replicate 3	133
replicate 1	272		replicate 1	108
replicate 2	245		replicate 2	124
replicate 3	240		replicate 3	173
replicate 1	288		replicate 1	154
replicate 2	255		replicate 2	151
replicate 3	321		replicate 3	104
replicate 3	297		replicate 3	135
avarage	**261,7**		avarage	**134,6**
st. dev	**31,8470302**		st. dev	**22,2870166**

OXIDATIVE STRESS				
48h				
Relative fluorescence in 150ul cell suspension				
	replicate 1			
BW25113	14185,25	14622,25	15921,25	15075,25
BW25113-ΔwrbA	18492,25	18524,25	18737,25	18970,25
	replicate 2			
	17079,25	17266,25	16941,25	17325,25
	11996,25	12041,25	12005,25	12278,25
	replicate 3			
	16314,25	17395,25	17345,25	17253,25
	18698,25	18536,25	18597,25	20602,25
	n. cells in 150 ul			
	3,0536E+15			
	8,247E+14			
Relative fluorescence/cells				
	replicate 1			
BW25113	4,6455E-12	4,7886E-12	5,214E-12	4,9369E-12
BW25113-ΔwrbA	2,2423E-11	2,2462E-11	2,272E-11	2,3003E-11
	replicate 2			
	5,5932E-12	5,6544E-12	5,548E-12	5,6738E-12
	1,4546E-11	1,4601E-11	1,4557E-11	1,4888E-11
	replicate 3			
	5,3427E-12	5,6967E-12	5,6803E-12	5,6502E-12
	2,2673E-11	2,2476E-11	2,255E-11	2,4982E-11
	Average	sd	err%	
	5,3687E-12	3,8279E-13	7,13014834	
	2,0157E-11	4,1264E-12	20,4715673	
60h				
Relative fluorescence in 150ul cell suspension				
	replicate 1			
BW25113	19740,25	20068,25	19980,25	
BW25113-ΔwrbA	22077,25	21726,25	21589,25	21622,25
	replicate 2			
	20038,25	20156,25	20301,25	20271,25
	20686,25	20681,25	20631,25	20768,25
	replicate 3			
	22960,25	22866,25	22889,25	21213,25
	22537,25	22578,25	22534,25	21883,25
	n. cells in 150 ul			
	6,5445E+15			
	7,4364E+17			
Relative fluorescence/cells				
	replicate 1			
BW25113	3,0163E-12	3,0664E-12	3,053E-12	
BW25113-ΔwrbA	2,9688E-14	2,9216E-14	2,9032E-14	2,9076E-14
	replicate 2			
	3,0618E-12	3,0799E-12	3,102E-12	3,0974E-12
	2,7817E-14	2,7811E-14	2,7743E-14	2,7928E-14
	replicate 3			
	3,5083E-12	3,4939E-12	3,4975E-12	3,2414E-12
	3,0306E-14	3,0362E-14	3,0302E-14	2,9427E-14
	Average	sd	err%	
	3,2016E-12	1,9962E-13	6,23494873	
	2,9059E-14	1,021E-15	3,51347041	

CATALASE ACTIVITY					
BW25113 48h (catalase activity U)					
	repl. 1	repl. 2	repl 3	avarage	st. dev
HPII	113	133	107	118	14
HPI	251	179	297	242	59
BW25113 60h (catalase activity U)					
	repl. 1	repl. 2	repl 3	avarage	st. dev
HPII	433	456	387	425	35
HPI	45	108	192	115	74
BW25113-ΔwrbA 48h (catalase activity U)					
	repl. 1	repl. 2	repl 3	avarage	st. dev
HPII	283	324	272	293	27
HPI	237	317	291	282	41
BW25113-ΔwrbA 60h (catalase activity U)					
	repl. 1	repl. 2	repl 3	avarage	st. dev
HPII	598	632	580	603	26
HPI	121	164	250	178	66

SUSCEPTIBILITY TO BIOCIDES				
BW25113 (log10 reduction)				
	repl 1	repl 2	avarage	st. dev
48h_HP	0,70733357	0,6392018	0,67326768	0,04817644
48h_HP+Gl	0,68557018	0,5658763	0,62572324	0,08463636
48h_CBA	1,16154554	1,70614859	1,43384707	0,38509251
48h_CBA+Gl	0,77579095	0,65259	0,71419047	0,08711623
60h_HP	0,65851719	0,39858676	0,52855198	0,18379857
60h_HP+Gl	1,20011624	1,30623119	1,25317372	0,0750346
60h_CBA	1,11367166	0,9431405	1,02840608	0,12058374
60h_CBA+Gl	0,99192237	0,6925549	0,84223864	0,21168477
BW25113-ΔwrbA (log10 reduction)				
	replicate 1	replicae 2	avarage	st. dev
48h_HP	2,99323577	3,10389613	3,04856595	0,07824869
48h_HP+Gl	1,68052002	1,4531541	1,56683706	0,16077198
48h_CBA	1,66171645	1,51373537	1,58772591	0,10463843
48h_CBA+Gl	1,3022347	0,96642417	1,13432943	0,2374539
60h_HP	1,79034139	1,98871899	1,88953019	0,14027414
60h_HP+Gl	1,34207383	1,36759599	1,35483491	0,01804689
60h_CBA	0,93029227	0,98871899	0,95950563	0,04131393
60h_CBA+Gl	0,95931291	0,88226366	0,92078828	0,05448205

**NADPH:*p*-BQ oxidoreductase activity (U/mg) of *E. coli* crude extracts**					
	replicates	**BW25113**		replicates	**BW25113-Δ*wrb*A**
	1_1	25,48		1_1	5,83
	1_2	25,61		1_2	5,45
	1_3	20,23		1_3	6,23
	1_4	24,92		1_4	7,19
	2_1	19,88		2_1	6,91
	2_2	21,53		2_2	9,22
	2_3	17,42		2_3	9,61
	3_1	20,40		2_4	11,49
	3_2	22,03		3_1	6,00
	3_3	23,96		3_2	6,38
	3_4	22,71		3_3	5,23
	3_5	21,67		3_4	5,63
				4_1	4,93
				4_2	5,89
				4_3	4,42
				4_4	5,49
				4_5	5,67
				5_1	5,54
				5_2	4,98
				5_3	10,58
				5_4	8,03
				5_5	5,47
				6_1	5,91
				6_2	5,09
				6_3	6,29
				6_4	5,26
				6_5	5,68
average		22,15			6,46
sd		2,51			1,79
Each datum arise from an independent assay reaction					

**NADPH:DCPIP oxidoreductase activity (U/mg) of *E. coli* crude extracts**					
	replicates	**BW25113**		replicates	**BW25113-Δ*wrb*A**
	1_1	2,27		1_1	1,49
	1_2	2,62		1_2	2,38
	1_3	3,22		1_3	1,28
	2_1	3,12		2_1	2,05
	2_2	5,54		2_2	2,01
	3_1	2,86		3_1	0,08
	3_2	3,33		3_2	1,43
	3_3	4,23		3_3	1,59
	3_4	3,00		4_1	2,17
				4_2	2,53
				4_3	2,04
				4_4	1,95
				5_1	1,60
				5_2	1,22
				5_3	1,52
				5_4	1,17
				6_1	1,57
				6_2	1,55
				6_3	1,57
				6_4	1,92
average		3,35			1,66
sd		0,98			0,53
